# Ferroptosis: emerging roles in lung cancer and potential implications in biological compounds

**DOI:** 10.3389/fphar.2024.1374182

**Published:** 2024-05-09

**Authors:** Qiuran Liang, Yuehui Wang, Yili Li, Jinyan Wang, Chuanbo Liu, Yicong Li

**Affiliations:** ^1^ The Second Clinical Hospital of Beijing University of Chinese Medicine, Beijing, China; ^2^ Dongfang Hospital of Beijing University of Chinese Medicine, Beijing, China

**Keywords:** ferroptosis, lung cancer, biological compound, resistance, nanotechnology

## Abstract

Lung cancer has high metastasis and drug resistance. The prognosis of lung cancer patients is poor and the patients’ survival chances are easily neglected. Ferroptosis is a programmed cell death proposed in 2012, which differs from apoptosis, necrosis and autophagy. Ferroptosis is a novel type of regulated cell death which is driven by iron-dependent lipid peroxidation and subsequent plasma membrane ruptures. It has broad prospects in the field of tumor disease treatment. At present, multiple studies have shown that biological compounds can induce ferroptosis in lung cancer cells, which exhibits significant anti-cancer effects, and they have the advantages in high safety, minimal side effects, and less possibility to drug resistance. In this review, we summarize the biological compounds used for the treatment of lung cancer by focusing on ferroptosis and its mechanism. In addition, we systematically review the current research status of combining nanotechnology with biological compounds for tumor treatment, shed new light for targeting ferroptosis pathways and applying biological compounds-based therapies.

## 1 Introduction

Lung cancer is a prevalent malignant neoplasm affecting the respiratory system. In 2022, lung cancer is estimated to account for 13% of new cancer diagnoses in the United States and 22% of all cancer-caused deaths. Although the mortality rate of lung cancer continues to decline with the decrease in the number of smokers and the early rational diagnosis and treatment, lung cancer is still the highest fatality rate of cancer. Due to the fact that smokers are more common among males, the number of deaths of lung cancer in males is higher than in females (Siegel et al., 2023). Therefore, improving the clinical efficacy and prognosis of lung cancer treatment, seeking more effective intervention targets and mechanisms, and exploring more advantageous treatment plans are urgent issues that need to be addressed.

Ferroptosis was first proposed by Dixon et al. in Cell Magazine in 2012. It is a regulated form of cell death that differs from other programmed deaths like morphology, biochemistry and regulatory mechanism (Dixon et al., 2012). Ferroptosis is based on divalent iron, characterized by the accumulation of iron-dependent polyunsaturated fatty acid peroxides, leading to membrane damage and cell lysis (Riegman et al., 2020; Du et al., 2023). The occurrence of ferroptosis is the result of various metabolic imbalances, and the disruption of metabolic homeostasis is related to tumors, inflammation, nerve damage, ischemic organ damage, etc. (Liang et al., 2022; Li et al., 2023; Ye et al., 2023). Ferroptosis regulators as promising biomarkers and therapeutic targets associated with tumor immune infiltration are shown to have accurate diagnostic and prognostic predictive performance with superior specificity and sensitivity (Tang et al., 2021a). Therefore, in-depth researches on ferroptosis have broad prospects in the field of tumor disease treatment.

Anti-cancer drugs mostly work by inducing tumor cell apoptosis, but long-term and high-dose use may lead to resistance to drugs that induce apoptosis, resulting in poor therapeutic effects. Previous studies have shown that ferroptosis is closely related to the occurrence of tumors. Due to the high metabolic levels, cancer cells require more iron during production process compared with normal cells (Hassannia et al., 2019). Also, the imbalance in iron metabolism is able to increase the risk of cancer and promote tumor growth, making them more sensitive to ferroptosis. As a result, ferroptosis is considered as an effective mean of treating with tumors (Tsoi et al., 2018), providing new directions for the development of anti-cancer drugs. It has been continuous reports that biological compounds play a potential anti-tumor role in regulating ferroptosis, which has the advantages in high safety, small side effects, and less susceptibility to drug resistance (Yan et al., 2021). This review will further explore the therapeutic targets and pathways of ferroptosis, analyze the mechanism of ferroptosis, and summarize the existing biological compounds targeting ferroptosis in order to provide theoretical basis for further research on ferroptosis.

## 2 The mechanism of ferroptosis

**FIGURE 1:**
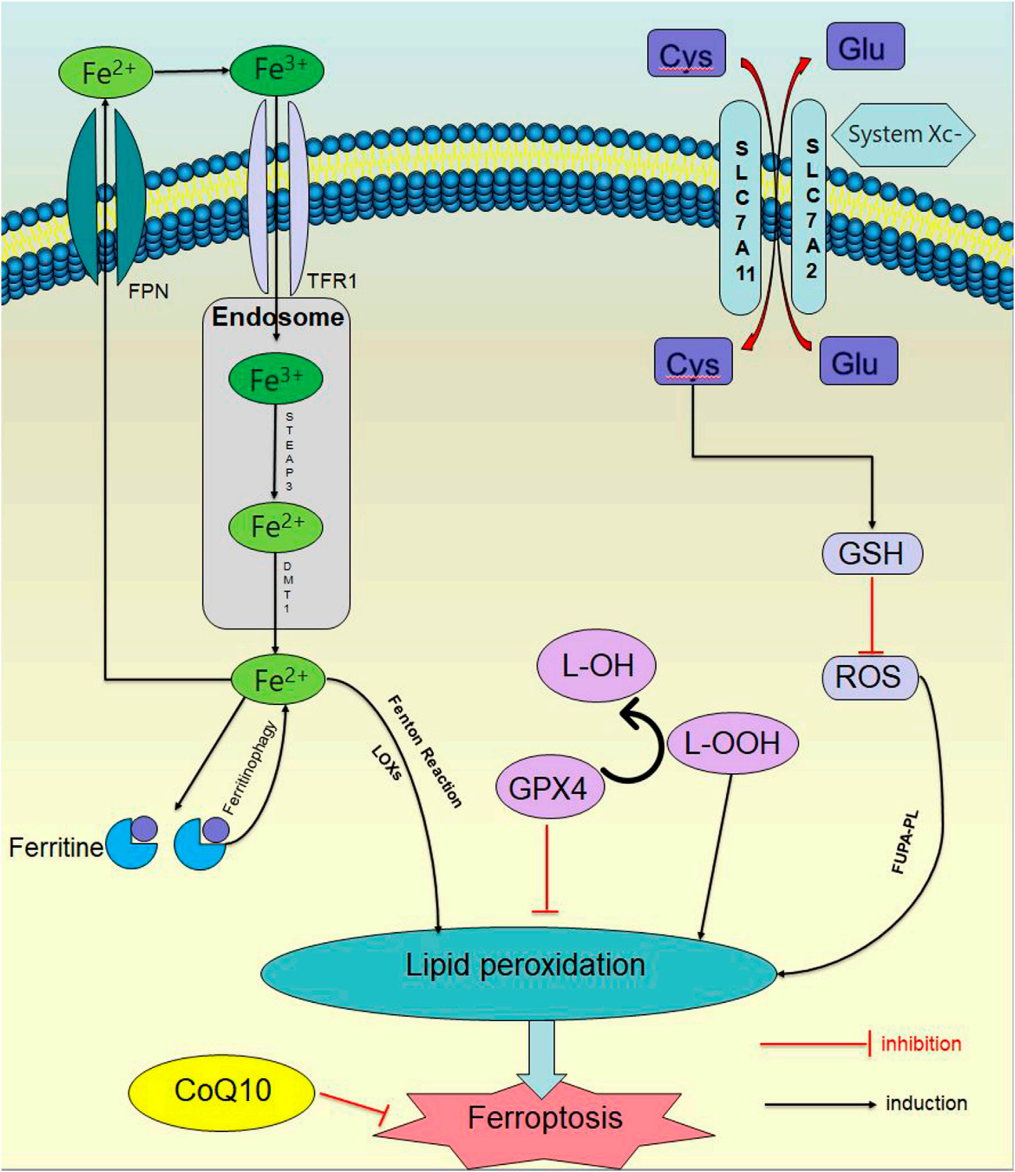
Ferroptosis signaling pathway. In the case of metabolic disorders, iron, lipids, and ROS) cannot maintain normal cellular function. Iron affects lipid peroxidation during ferroptosis by producing ROS and activating iron containing enzymes. The excessive production of lipid ROS promotes damage and destruction of cell membranes, leading to cell death.

Ferroptosis is controlled and executed by a comprehensive and precise signal network, including genetic, transcriptional, post-transcriptional, and post-translational level (Tang et al., 2021b). Ferroptosis is a programmed cell death distinct from apoptosis, necrosis and autophagy, which is the result of various metabolic imbalances. In the case of metabolic disorders, iron, lipids, and reactive oxygen species (ROS) cannot maintain normal cell function. Iron affects lipid peroxidation during ferroptosis by producing ROS and activating iron-containing enzymes (such as arachidonic acid lipoxygenase (ALOXs)). The mass production of lipid ROS promotes cell membrane damage and destruction, leading to cell death (Zhou et al., 2020; Zheng et al., 2023). Ferroptosis has two characteristics: firstly, morphological features, include cell membrane rupture, mitochondrial shrinkage, mitochondrial ridge reduction or even disappearance, increased mitochondrial membrane density, outer membrane rupture, and normal nuclear morphology, but lacking chromatin agglutination; secondly, biological characteristics, include plenty of ROS production, aggregation of ferrous ions, activation of mitogen-activated protein kinase system (MAPK), glutathione (GSH) reduction, and cysteine-glutamate antiporter system (System Xc-), etc (Jiang et al., 2021a); (Figure 1).

### 2.1 Iron metabolism disorder

Under physiological conditions, extracellular iron exists in the form of trivalent iron (Fe^3+^), binds to transferrin receptor 1 (TFR1) and enters the cell. After entering the nucleus, it is immediately reduced to divalent iron ions (Fe^2+^) under the catalysis of iron reductase prostate six transmembrane epithelial antigen 3 (STEAP3). Afterwards, Fe^2+^ is transported to the cytoplasm through the endosomal membrane under the action of divalent metal transporter 1 (DMT1), and form an unstable iron pool. The excess iron is transported to the extracellular space through the iron transporter protein, or stored in ferritin and reacted with intracellular H_2_O_2_ to generate highly active hydroxyl radicals (•OH) under slightly acidic conditions, which is known as the Fenton reaction (Fe^2+^ + H_2_O_2_→Fe^3+^ + OH^−^ +·OH). •OH then reacts with intracellular liposomes to generate lipid peroxidation (LPO) (Sutton and Winterbourn, 1989; Liu et al., 2018a). At the same time, it has been reported that ferritin-bound iron can be mobilized through selective autophagy to degrade ferritin (which is known as ferritinophagy). In other words, when intracellular iron is deficient, the ferritinophagy receptor NCOA4 (nuclear receptor coactivator 4) is stabilized, interacting with ferritin and delivering ferritin to the autophagosomes. NCOA4 is engulfed by lysosomal degradation and releases iron, then the sensitivity to ferroptosis is increased (Shan et al., 2023; Wu et al., 2023).

### 2.2 Abnormal antioxidant mechanism

Multiple antioxidants are present in the cell. GSH is the main intracellular antioxidant, and it is also a cofactor for the normal function of selenase glutathione peroxidase 4 (GPX4). The System Xc-on the cell membrane is an important part of keeping the homeostasis of the cellular antioxidant system, consisting of two parts: a light chain (xCT, transcribed by SLC7A11) and a heavy chain (4F2, transcribed by SLC3A2) (Xu et al., 2023a), which can exchange intracellular glutamate with extracellular cysteine (Parker et al., 2021). After cystine enters the cell, it is reduced to cysteine by nicotinamide adenine dinucleotide phosphate (NADPH). Due to the limited concentration of cysteine in cells, cysteine is considered as a rate-limiting precursor for GSH synthesis, which further synthesizes GSH with glycine and glutamate acid to reduce intracellular ROS.

When System Xc - is inhibited, decrease in intracellular GSH synthesis leads to a significant accumulation of ROS, causing an imbalance in the cellular antioxidant system. When ROS accumulates in large quantities, it reacts directly with polyunsaturated fatty acid phospholipids (PUFA-PL) on the cell membrane to form lipid hydroperoxides, which in turn forms LPO and initiates ferroptosis. In addition, lipid hydroperoxides can also be directly catalyzed by the combined action of lipoxygenases (LOXs) and Fe^2+^.

Besides, GPX4 is mainly involved in the redox reaction of ferroptosis (Ingold et al., 2018). GPX4 is selenium protein, which is the only enzyme that directly reduces lipid hydroperoxides in biofilms, catalyzing the reduction of lipid hydroperoxides, converting toxic lipid hydroperoxides (L-OOH) to non-toxic lipid alcohols (L-OH) in the lipid bilayer (Ursini et al., 1982), reducing LPO and maintaining cellular redox homeostasia. Once the production of the (e)ine/GSH/GPX4 axis is inhibited, lethal amounts of LPO will accumulate, resulting in disruptions such as cell membrane rupture, cell swelling, mitochondrial rupture and leading to ferroptosis (Li and Li, 2020; Shan et al., 2023). The expression of GPX4 is regulated by GSH and selenium, whose catalytic requirement is fulfilled by replacing cysteine and two electrons with selenium supplied by GSH, and GPX4 is inactivated while GSH is consuming.

The pathway of ferroptosis is influenced by various enzymes as well. Lipoxygenases (LOXs), non-heme iron-dependent dioxygenases, can selectively act on polyunsaturated fatty acids (PUFAs) and lipid-containing PUFAs in oxidative biofilms, thereby increasing the likelihood of ferroptosis. LOXs mainly induce ferroptosis through cysteine deprivation, rather than depletion of GPX4. Cytochrome p450 oxidoreductase (POR) affects iron metabolism by removing hydrogen from polyunsaturated fatty acids, or by reducing Fe^3+^ to Fe^2+^ for electron transfer, to accelerate the circulation between Fe^3+^ and Fe^2+^ to promote ferroptosis (Zou et al., 2021). Lipoxygenase isoenzymes and p450 oxidoreductase can catalyze PUFA-PL to produce PLOOH and induce ferroptosis (Ni and Li, 2024).

Another study has found that nuclear factor erythroid 2-related factor (Nrf2) is an important antioxidant transcription factor, which plays a key role in oxidative defense by regulating the expression of various antioxidant factors. At the same time, Nrf2 is also a crucial regulatory factor for maintaining oxidative homeostasis in oxidative damage induced by the ROS production factor, NADPH oxidase 4 (NOX4). Nrf2 is a negative regulator of ferroptosis as well, which can regulate the expression of several key genes involved in iron concentration by activating the GSH transport system, improving ferritin autophagy, regulating mitochondrial activity and lipid peroxidation (Hou et al., 2024).

### 2.3 Peroxidation of membrane phospholipids

Unrestricted peroxidation of phospholipids is another vital mechanism that promotes ferroptosis. Lipid metabolomics has shown that polyunsaturated fatty acids such as arachidonic acid (AA) or adrenergic acid (AdA) is the most easily oxidized lipids during ferroptosis and are regulated by three synthases. Acyl-CoA synthase long-chain family member 4 (ACSL4) catalyzes the conversion of AA or AdA to AA-CoA and AdA-CoA. Subsequently, lysine phosphatidylcholine acyltransferase 3 (LPCAT3) esterifies it to phosphatidylethanolamine (PE) to form AA-PE and AdA-PE, and finally oxidized to PE-AA-OOH and PE-AdA-OOH through polyunsaturated fatty acid lipoxygenase 15 (ALOX15) (Jiang et al., 2024).

The sensitivity of cells to ferroptosis is mainly determined by the unsaturation exhibited by the phospholipid bilayer. Biological cell membranes and organelle membranes are particularly vulnerable to ROS because they are rich in PUFA (Viswanathan et al., 2017) that is easily damaged by oxidation, then the lipid bilayer is destroyed and membrane function is affected, which are becoming drivers for ferroptosis. Phospholipids peroxidation begins with the lipid bilayer containing PUFA-PL, removing two hydrogen atoms (located between two carbon-carbon double bonds) from the diallyl group of PUFA-PL’s unsaturated acyl group, and forming a carbon-centered phospholipid radical that reacts with oxygen to produce a phospholipid hydrogen peroxide radical. Then two hydrogen atoms are removed from polyunsaturated fatty acid (PUFA) and form phospholipid hydroperoxide (PLOOH). If PLOOH is not reduced to corresponding alcohols by GPX4, these substances will continue to oxidize PUFA-PL to produce more PLOOH (Conrad and Pratt, 2019), triggering the accumulation of lethal lipid ROS (Ma et al., 2022), causing organelle and/or cell membrane rupture (Jiang et al., 2021b), and inducing ferroptosis. On the contrary, monounsaturated fatty acid (MUFA), such as saturated fat palmitic acid (SFA), is incorporated into phospholipids in an ACSL3 dependent manner through stearoyl CoA desaturase-1 (SCD1). This binding interferes with the formation of PUFA phospholipids and protects cells from cell death caused by ferroptosis (Xiang et al., 2024).

Moreover, a study in 2019 (Doll et al., 2019) showed that the FSP1-CoQ10-NAD(P)H pathway existed as an independent parallel system, which inhibited ferroptosis by producing the antioxidant CoQ10 on the plasma membrane to capture phospholipid peroxidation free radicals, and collaborated with GPX4 and glutathione (GLU) to inhibit phospholipid peroxidation and ferroptosis. Later, DHODH, located in the inner mitochondrial membrane, reduced CoQ10 to panthenol and played a role in ferroptosis inhibition. Thus, several anti-ferroptotic pathways, including SLC7A11/GPX4, FSP1/CoQ, GCH1/BH4, and mitochondria DHODH/CoQ, were established as LPO defending systems to prevent ferroptosis (Gao et al., 2022).

The acyl chain saturation of membrane phospholipids is strongly correlated with the ferroptotic sensitivity of cells, but the influence of specific phospholipid categories on ferroptosis remains obscure. Some scholars (Zhu et al., 2023)have found that phosphatidylcholine (PC) reduction strongly induced ferroptosis, but they excluded the possibility that the sensitivity to ferroptosis is caused by elevated PUFA levels. Meanwhile, PC was required in the construction of new membranes, which made cells are more sensitive to PC deficiency than other tissues. Therefore, it was feasible to induce ferroptosis by simultaneous treatment with canonical ferroptotic inducers and PC synthesis inhibitors.

## 3 Clinical researches progress of targeting ferroptosis in the treatment of tumors

Ferroptosis has dual properties in the progression of cancer: on the one hand, ferroptosis can help tumor cells evade immune surveillance and reduce the effectiveness of early tumor treatment; on the other hand, it can resist tumor drug resistance, increase sensitivity to radiation, and assist in enhancing immunotherapy (Li et al., 2024). Targeting ferroptosis has been clinically used to treat tumors, whose mechanisms mainly include inhibiting System Xc-, inhibiting/degrading/inactivating GPX4, consuming coenzyme Q10, and inducing lipid peroxidation through peroxides, iron, or PUFA overload (Bian et al., 2024). Diffuse large B-cell lymphoma (DLBCL) is a heterogeneous lymphoid malignancy. It has found that (Zhang et al., 2019; Schmitt et al., 2021; Hong et al., 2022) dimethyl fumarate (DMF) consumes GSH and inhibits NF-κB and JAK/STAT survival signals; APR-246 promotes the binding of repaired p53 mutants to target genes and their transcription factors; Imidazolone Erastine (IKE) is a potent and metabolically stable systemic Xc-inhibitor and ferroptosis inducer by consuming GSH. All of these possess the ability to cause ferroptosis and treat DLBCL. Radiation therapy (RT) is the main treatment method for tumors, but it inevitably damage to normal cells. Ning et al. (2024) found that some plant extracts, exosomes, and plasmids exhibited the repair of radiation-induced hematopoietic and skin damage by inhibiting ferroptosis; Melatonin promoted the binding of Nrf2 and pyruvate kinase isoenzyme M2 (PKM2), which reduced iron concentration and inhibited ferroptosis to alleviate radiation-induced hippocampal neuronal death. Yue et al. (2024)summarized that various anti-tumor drugs such as cisplatin, paclitaxel, capecitabine, apatinib and artemisinin could induce ferroptosis in gastric cancer (GC) cells. And even dexmedetomidine (DEX), levobupivacaine and other local anesthetics had potential value in the treatment of GC due to their ability in regulating ferroptosis and inhibiting GC ell proliferation.

## 4 Biological compounds intervene in ferroptosis in lung cancer

At present, most studies on inducing ferroptosis mainly focus on western medicine pathways, including targeted dysregulation of iron metabolism, targeted reduction-oxidation pathways, targeted lipid metabolism pathways and other strategies. However, to date, there has been no specific summary of the progress about biological compounds curing lung cancer. Natural plants are a rich source of bioactive ingredients and extracts, with various biological activities, including anti-cancer properties, antioxidation, immunoregulation, and antibacterial effects. Natural compounds have always been of great pharmacological significance, especially in chemoprevention, since they have high availability and affordability. Biological compounds can be performed as substitutes or adjuncts for other anti-tumor therapies for cancer treatment, which increase the sensitivity of tumor cells to radiotherapy and chemotherapy, and even reversing the MDR response to chemotherapy. They can also regulate tumor related immune responses and improve the prognosis of anti-tumor therapy. The latest researches on the promotion of ferroptosis in lung cancer by biological compounds show that they mainly block the progression in the G2/M phase and cause a decrease in the number of G0/G1 and S phase cells (Chen et al., 2020a; Luo and Xu, 2022; Wu et al., 2022; Zhou et al., 2023a). Several key factors, such as glutathione peroxidase 4 (GPX4), reactive oxygen species (ROS), glutathione (GSH), solvecarrierfamily7, and (ionicamino acidtransporter, y + system) member11 (SLC7A11), play important roles in this process. The research progress on the induction of ferroptosis by biological compounds in the treatment of lung cancer is listed in Table 1.

**TABLE 1:** Biology compounds as ferroptosis regulator in lung cancer.

Biological compounds	Resourse	Activity	Mechanism	Target spot/Pathway	Reference
Erianin	Dendrobium chrysotoxum Lindl	H460 and H1299	ROS and lipid peroxidation accumulation, GSH depletion, the expression of negative regulatory proteins (GPX4, CHAC2, SLC40A1, SLC7A11, and glutaminase) decreasing	-	Chen et al. (2020a)
Fascaplysin	Sponge	A549	Increased level of ROS and Fe^2+^, downregulation of iron related proteins, and enhancement of anti-PD-1 anti-tumor effect	Wnt/β-catenin pathway	Luo and Xu (2022)
α-hederin	Taxus chinensis	A549 and PC9	Disrupting the GSH redox system mediated and increasing the sensitivity to cisplatin	GSS/GSH/GPX2 pathway	Wu et al. (2022)
Timosaponin AIII	Anemarrhena Rhizome	H1299, A549, SPC-A1, LLC	Release of ROS and accumulation of iron, production of MDA and depletion of GSH	HSP90	Zhou et al. (2023a)
Curcumin	Curcuma	A549	Inhibition of GPX4 and FSP1 expression	GSH-GPX4,FSP1-CoQ10-NAPH Pathways	Zhou et al. (2023b)
6-gingerol	Ginger	A549	Reducing the concentration of autophagosom-es, ROS, and iron in USP14	-	Tsai et al. (2020)
β- elemene + erlotinib	Curcuma	H1975, H1650, H1819	Decreasing ferroptosis negative regulatory proteins, increasing ROS, lipid peroxidation levels	-	Xu et al. (2023b)
Solasonine	Nightshade	Calu-1, A549	Inhibiting the expression of SLC711 and GPX4, hindering mitochondrial function and exacerbating redox imbalance	-	Zeng et al. (2022)
Hedyotis diffusa	Hedyotis	A549, H1975	Regulating VDAC2/3 activity by promoting Bax via inhibiting Bcl2	VDAC2/3	Huang et al. (2022)
Scoparone/6,7-dimethoxycoumarin	Binhao/Chenhao	A549, H1299, PC-9	Upregulation of lipid peroxidation, ROS, and iron levels	ROS/JNK/SP1/ACSL4	Shen et al. (2023a)
Bufotalin	Toad	A549	Increasing intracellular Fe^2+^, accelerating GPX4 degradation and promoting lipid peroxidation	-	Zhang et al. (2022a)
Andrographolide	Andrographis Paniculata	H460, H1650	Elevated levels of ROS, GSH, malondialdehyde (MDA), intracellular iron content and lipid ROS reducing GSH accumulation	-	Jiaqi et al. (2023)
Diplacone	Paulownia tomentosa	A549	Inducing lipid peroxidation and ATF3 expression, increasing ROS production	-	Oh et al. (2023)
tanshinol A	Alvia miltiorrhiza	NCI-H1299	Inducing ROS generation	-	Liu et al. (2020)
Curcumenol	Curcuma	CCD19, H1299, H460	lncRNA H19 and GPX4 decreased, FSLC7A11,HO-1 and transferrin expression increased	lncRNA H19/miR-19b-3p/FTH1 pathway	Zhang et al. (2021a)
Red ginseng polysaccharide	Ginseng	A549, MDA-MB-231	Inducing the release of lactate dehydrogenase (LDH), inhibiting GPX4 and accumulating ROS	-	Zhai et al. (2022)
Huaier aqueous extract	Huaier	NCI-H1299	Accumulating ROS	MAPK pathway	Tian et al. (2020)
Sinapine (SI)	Mustard Seed	A549, SK, and H661	Increasing Fe^2+^, lipid peroxidation and ROS	-	Shao et al. (2022)
Capsaicin	Pepper	A549 and NCI H23	Increasing total iron and Fe^2+^, reducing reduced glutathione and GPX4	SLC7A11/GPX4 pathway	Liu et al. (2022a)
Ophiopogonin B	Ophiopogonis	A549	MDA, GSH and concentrations of intracellular iron elevated, the expression of GPX4 decreased	-	Li et al. (2022a)
Artesunate (ART) and DHA	Artemisia	NCI-H1299, A549, LTEP-a-2, NCI-H23, NCI-H358	ROS stimulation	-	Zhang et al. (2021b)
DHA	Artemisia	LLC	Promoting iron ion enrichment and inhibiting GPX4, producing ROS and LPO	-	Li et al. (2022b)
Celastrol + erastin	Tripteryg-Ium wilfordii	HCC827, A540, H1299	Increasing generation of ROS	ATG5/ATG7 pathway, PINK1/Parkin pathway	Huang et al. (2021)
Camptothecin-Loaded and Manganese Dioxide-Coated Polydopamine Nanomedicine	Camptotheca acuminata	A549,LLC	Low expression of GSH levels and GPX4, accumulation of ROS and LPO	-	Su et al. (2022)
Realgar	Realgar	KRAS mutation H23,A549,H460, non KRAS mutation H1650	Elevating intracellular levels of Fe^2+^, ROS and GSH, reducing expression of GPX4 and SCL7A11	Ras/MAPK pathway	Liu et al. (2022b)
Sanguinarine (SAG)	Sanguinaria canadensis Linn	A549,H3122	Fe^2+^ and ROS increased, while GSH decreased, the protein stability of GPX4 reduced	-	Xu et al. (2022)
Isoorientin (IO)+ Cisplatin (DDP)	Bamboo leaf	A549	The concentration of intracellular iron, MDA and ROS increased, while glutathione decreased	SIRT6/Nrf2/GPX4 pathway	Feng et al. (2023a)
Ginkgetin + Cisplatin (DDP)	Ginkgo biloba leaves	A549,NCIH460,SPC-A-1 cell lines	Increasing lipid peroxidation, reducing expression of SLC7A11 and GPX4	Nrf2/HO-1 pathway	Lou et al. (2021)
dihydroisotanshinone I (DT)	danshen	A549,H460,IMR-90	Blocking GPX4 protein expression and increasing ROS accumulation	Nrf2 pathway	Wu et al. (2021)
Bufotalin (BT)	Toad	A549	Inhibiting the expression of GPX4 protein	-	Zhang et al. (2022a)
Shikonin	Lithospermum erythrorhizon	SBC-2 and H69	Increase in total ROS and lipid ROS, decrease in GSH levels	c-myc/HDAC1 axis	Qian et al. (2023)
Gigantol	Dendrobium genus	H460, A549	Inhibiting GSH synthesis and GPX4 activity, and disrupting the REDOX balance *in vivo*	SLC7A11-GPX4 signaling axis	Chen et al. (2023)

Biological compounds can be classified into different categories based on their chemical composition, containing polyphenols, terpenoids, alkaloids, flavonoids, etc., all of which have an undeniable ability to induce ferroptosis in tumors. In 2015, Professor Tu Youyou was awarded the Nobel Prize in Medicine for extracting artemisinin from the plant Artemisia annua as an anti-malaria drug, which was proved to be a promising drug for treating various diseases, embodying cancer. Further researches have shown that artemisinin and its derivatives can stimulate the ferroptosis cell pathway, containing down-regulating GPX4 expression, increasing ROS and intracellular iron production. There are some natural compounds defined as ferroptosis inducers, such as Ferroptoside (Llabani et al., 2019), Erianin (Luo and Xu, 2022) and Wogonin (Liu et al., 2023a). Ferrotocide, a diterpenoid natural product, is a thioredoxin inhibitor which inducs ferroptosis of breast cancer by inactivating thioredoxin (a cell antioxidant enzyme). Erianin is a natural product derived from Dendrobium chrysotoxum Lindl, and Ca^2+^/CaM signaling is a key medium of ferroptosis erianin causing. Wogonin, which is extracted from the root of Scutellaria baicalensis Georgi, significantly inhibited the proliferation of pancreatic cancer cells and induced ferroptosis through Nrf2/GPX4 axis.

In addition, although some natural compounds with biological activity like curcumin are shown to activate ferroptosis, they can also be used as ferroptosis inhibitors for tissue damage treatment. Curcumin is the main phenolic pigment extracted from turmeric (the powdery rhizome of turmeric), which inhibits the expression of GPX4 and FSP1 in lung cancer cells (Zhou et al., 2023b), disrupting GPX4 activity in glioblastoma cells (Chen et al., 2020b) and stimulating ferroptosis. On the contrary, curcumin has strong anti-inflammatory and antioxidant properties, being able to eliminate free radicals such as superoxide anions and hydroxyl groups to neutralize ROS. It has been reported that curcumin has ability to inhibit lipid peroxidation in renal epithelial cells, thereby preventing the cytotoxic effects of hydrogen peroxide (Ahangarpour et al., 2019). Thus, more research is needed to demonstrate the biochemical and/or cellular conditions of the action modes of these biological compounds.

Ferroptosis has been recognized as the pathogenic mechanism of many diseases, such as neurodegenerative diseases, ischemia-reperfusion (I/R) injury, etc. And some natural compounds can cure diseases depending on ferroptosis. For example, Hinokitiol (β- Thujaplicin, HIK) is a natural monoterpene small molecule compound with antibacterial, anti-inflammatory, anti-tumor and various biological activities. Hinokitiol is capable to alleviate excessive glutamate to induce ROS, lipid peroxidation, and Fe^2+^accumulation in neuronal cells and relieve brain tissue damage (Tang et al., 2023). Quercetin (QRC) is absorbed through the intake of diverse fruits, vegetables, and legumes, which upregulates GPX4 and FTH1 and downregulate ACSL4 to inhibit the production of lipid peroxides and ferroptosis. It exerts neuroprotective effects, and demonstrates the therapeutic potential for ischemic stroke (Peng et al., 2023). Baicalin supresses oxidative stress by reducing ROS and protects GPX4 from membrane lipid peroxidation, thereby it curbs ferroptosis and plays a neuroprotective role in cerebrovascular diseases, neurodegenerative diseases, and other central nervous system diseases (Yang et al., 2023).

## 5 Biological compounds improve lung cancer drug resistance through ferroptosis

The therapy for lung cancer mainly involves surgery combined with radiotherapy and chemotherapy. Cisplatin, gefitinib, osimertinib, etc. are well-known chemotherapy drugs used to treat tumors. However, acquiring resistance to chemotherapy is a major clinical issue, and the mechanism of this resistance is still unknown. So overcoming resistance is the main challenge. Ferroptosis is a special form compared with other forms of cell death, caused by the accumulation of iron-related lipid ROS. Cancer cells are more iron-addicted than normal cells because iron is one of the essential elements in the body. Therefore, cancer cells require more iron in the production process, so that the imbalance of iron metabolism leads to increase the risk of cancer and facilitates tumor growth. By accelerating ferroptosis progress, the development of lung cancer can be suppressed, while the drug resistance and radiation resistance of lung cancer is also resisted to a certain extent (Mou et al., 2019). Studies have shown that achieving ferroptosis increases ROS levels in cells and the cytotoxicity of cisplatin (Golbashirzadeh et al., 2023). Nevertheless, osimertinib-resistant cells are prone to ferroptosis due to raised accumulation of ferrous ions (Konishi et al., 2023). Therefore, a deeper understanding of the process of ferroptosis may guide the discovery of new therapies to overcome tumor resistance.

Biological compounds can be used together with chemotherapy drugs to enhance the sensitivity of the body to chemotherapy drugs and slow down the resistance process by ferroptosis of tumor cells. Feng et al. (2023a) found that the combination of isoorientin (IO) and DDP noticeably decreased the survival ability of drug-resistant cells, regulated ferroptosis and reversed\ drug resistance in lung cancer cells by controlling the SIRT6/Nrf2/GPX4 signaling pathway. Lai et al. (2023) observed that the combination therapy of dihydroartemisinin (DHA) and gefitinib had a better inhibitory effect on lung adenocarcinoma than gefitinib alone. Therefore, ROS is the key factor for DHA to resist the proliferation of drug-resistant lung adenocarcinoma cells and trigger ferroptosis. Li et al. (2022c) used D-borneol and cisplatin to treat non-small cell lung cancer (NSCLC) cells resistant to cisplatin and discovered that it could shrink tumor volume and weight, enhance anti-tumor effect, promote ROS accumulation, stimulate ferritin uptake mediated by nuclear receptor co activator 4(NCOA4), and upregulate PRNP and downregulate PCBP2 to adjust intracellular iron transport to achieve ferroptosis. Yu et al. (2023a) identified a natural NQO1 substrate, 2-methoxy-6-acetyl-7-methyljuglone (MAM), which has potent anti-cancer effects. MAM activates and binds to quinone oxidoreductase 1 (NQO1) which triggers ROS generation, unstable iron pool (LIP) expansion, and lipid peroxidation, activating NQO1-mediated ferroptosis to combat drug resistance.

Plenty of research has identified the crucial part of GPX4 in the susceptibility to drug-mediated ferroptosis in lung cancer, and targeting GPX4 can re-sensitize drug-resistant lung cancer to chemotherapy drugs (Zhang et al., 2022b; Cheng et al., 2023). Wu et al. (2022) have explored that the β-elemene integrated with erlotinib enhanced the cytotoxicity of erlotinib against EGFR mutated primary EGFR-TKI resistant NSCLC cells, and diminished the content of ferroptosis negative regulatory proteins (glutaminase, FTH1, Nrf2, Slc7a11 and GPX4), added ROS and lipid peroxidation levels, and accomplished anti-tumor effects through ferroptosis. In the bargain, Ni et al. (2023) found out that Manoalide (MA) enhanced the sensitivity of osimertinib-resistant lung cancer cells to osimertinib by inhibiting the NRF2-SLC7A11 axis and mitochondrial Ca^2+^ overload inducing -FTH1 pathway to activate ferroptosis.

## 6 Nano-drug delivery strategy for inducing ferroptosis in tumor

In recent years, emerging biomedical developments and technological innovations have opened up more possibilities for the effective treatment of cancer. The research on nano-drug delivery system has become an essential choice in cancer therapy. This system overcomes some drawbacks of direct-drug delivery and has significant advantages like improving water solubility, prolonging circulation time, enhancing tumor accumulation, increasing cell uptake and reducing toxicity. It has enormous potential in increasing therapeutic effects.

Nano-technology enables to deliver the active ingredients in sufficient concentrations to the desired site of action, and maintain the biological distribution and accumulation of therapeutic drugs at the desired target site. It avoids premature exposure to the biological environment during delivery, enhances its permeability, and heightens its ability to be absorbed by cells. A number of studies have shown that nanoparticles have the potential to scale up the solubility and stability of phytochemical extracts (Lawson and Gardner, 2005; Putnik et al., 2019). As a result, the use of nanotechnology with biological compounds has advantages in the utilization, which are considered as potential approaches (Lu et al., 2022). Table 2 presents the research on the use of nanotechnology combined with biological compounds to treat tumors through ferroptosis. At present, the biological effects of bioactive inorganic nanomaterials and metal ions have attracted widespread attention (Pei et al., 2023). Nano-carriers whose surfaces are designed to contain active ligands (cancer cell markers or antibodies) - binding structures, based on metals (such as systems carrying transferrin drugs), polymers (nanocapsules and nanospheres), or lipids (such as sulfur-containing glycoside nanoliposomes) carries ferroptosis inducers, which can be modified to target specific cancer cells and achieve anti-tumor effects (Neganova et al., 2022).

**TABLE 2:** Nano-drug delivery strategy for inducing ferroptosis in tumor cells.

Nanosystems	Composition	Mechanism	Reference
Camptothecin (CPT)	CPT-Loaded and Manganese Dioxide-Coated Polydopamine (PDA) Nanomedicine	Low expression of GSH levels and GPX4, accumulation of ROS and LPO	Su et al. (2022)
LDM	Inhalable biomineralized liposomes loaded with DHA and pH responsive CaP	Endoplasmic reticulum (ER) stress response centered on Ca^2+^burst enhances iron transport therapy in lung cancer. The Fenton-like reaction promotes intracellular ROS production	Lu et al. (2022)
Fe_3_O_4_-PGA-DHA and Fe_3_O_4_-PASP-DOX combination	Combination of Fe_3_O_4_ magnetic nanoparticles stabilized by polyglutamic acid loaded with DHA and Fe_3_O_4_ magnetic nanoparticles stabilized by polyaspartic acid loaded with DOX	Stronger cytotoxicity to different triple negative breast cancer cell lines, and more intracellular accumulation of ROS and lipid peroxide	Zhang et al. (2023b)
PMCs	Cyanobacteria and up-conversion nanoparticles encapsulated in alginate microcapsules	The development of lipid peroxidation, GPX4 inhibition, Fe^2+^release, and GSH reduction	Sun et al. (2023)
FeTPt@CCM	Integration of Fe^3+^, meso tetra (4-carboxyphenyl) porphyrin, and oxaliplatin prodrug into a MOF nanosystem (named FeTPt)	Activated by a Fenton-like reaction as well as a redox reaction between Fe^3+^ and glutathione and hydrogen peroxide to generate hydroxyl radical and oxygen	Zhang et al. (2023d)
FeGd-HN@Pt@LF/RGD_2_	A Fe_3_O_4_/Gd_2_O_3_ hybrid nanoparticle loaded with cisplatin (CDDP), combined with lactoferrin (LF) and RGD dimer (RGD2)	Fe^2+^, Fe^3+^, and CDDP are released from the body. Fe^2+^and Fe^3+^ directly participates in the Fenton reaction, while CDDP accelerates the Fenton reaction and produces ROS.	Shen et al. (2018)
Pt-FMO	A manganese deposition iron oxide nanoplatform loaded with cisplatin prodrug	Releasing manganese, iron ions, and platinum drugs in the tumor microenvironment, and catalyzing the Fenton reaction	Cheng et al. (2021)
Fe_3_O_4_-PLGA-Ce6	Nanosystem coated with poly (lactate-glycolic acid) (PLGA) containing iron oxide (Fe_3_O_4_) and Chlorin e6 (Ce6)	Releasing ferrous/trivalent iron ions that undergo Fenton reaction with excess hydrogen peroxide in the cell and producing •OH.	Chen et al. (2021)
AMSNs	Arginine (Arg) as a surface ligand for tumor targeting, a ferroptosis inducer of arginine manganese silicate nanobubbles (AMSNs)	Having highly efficient GSH depletion capacity, thereby inducing ferroptosis through GPX4 inactivation	Wang et al. (2018)
MnMoOx NPs	Manganese molybdate nanoparticles	Depletion of highly expressed GSH and inhibition GPX4 expression, inducing immune cell death	Lei et al. (2023)
TAF3-HMON-CuP3@PPDG	Tamoxifen (TAF), copper peroxide (CuP), organic silica nanoparticles (HMON)	Massive production of ROS to enhance ferroptosis therapy (FT)	Huang et al. (2023)
PMVL	Composed of vanadium oxide coordinated with tannic acid loaded with LND and disguised as tumor cell membrane modified with PD-L1 inhibitory peptide	Generating ·OH for lipid peroxide accumulation (VIV → VV) and consuming GSH to inactivate GPX4 (VV →VIV)	Zhang et al. (2023e)
CSO-SS-Cy7-Hex/SPION/Srfn	A self-assembled magnetic nano photosensitizer composite loaded with sorafenib	Inducing ferroptosis through redox reactions induced by high levels of ROS and GSH, causing tumor decomposition	Sang et al. (2019)
DOX-Fe(VI)@HMS-HE-PEG(DFHHP)	Integrating ferrate and doxorubicin into biocompatible hollow mesoporous silica	Releasing ferrate to react with GSH. GSH depletion inactivating GPX4 and inhibiting the reduction of lipid peroxides	Fu et al. (2021)
SRF@FeIIITA	Fe^3+^ ions and natural tannic acid (TA) spontaneously form nanoparticles on Sorafenib (SRF, ferroptosis promotor) nanonuclei	Allowing SRF release through mesh corona to inhibit GPX4 enzyme	Liu et al. (2018b)
SRF@Hb-Ce6	Hemoglobin (Hb) is linked to photosensitizer chlorin e6 (Ce6) and loaded with SRF	Ce6 producing cytotoxic 1O_2_ by laser irradiation of molecular oxygen carried by Hb and increasing the degree of lipid peroxidation	Xu et al. (2020)
siMCT4-PAMAM-PEG-TK-Fc@DEM	Ferrocene is conjugated on PEGylated polyamidoamine dendrimers, which is self-assemble with the diethyl maleate (DEM) and monocarboxylate transporter 4-inhibiting siRNA (siMCT4)	Activated by the elevated ROS levels, and DEM binding to GSH to disrupt GPX4-mediated antioxidant defense	Zhang et al. (2022c)
TKPFH NP	Using fluorinated polyethylene imine 1.8K (TKPF) as the core and hyaluronic acid (HA) as the shell to encapsulate TKPF NP	shGPX4 plasmids downregulates GPX4 and produces ROS and lipid peroxides, while GSH consumption indirectly inhibits GPX4 and further enhances ferroptosis	Yang et al. (2022)
MLP@DHA&Ce6	Modified manganese dioxide nanoshell on liposomes loaded with DHA and photosensitizer Ce6	Generating ROS via nanozyme-catalyzed CDT using DHA as a substrate, PDT through Ce6, and the Fenton reaction catalyzed by Mn^2+^ ions	Liu et al. (2023c)
GAN	Self-assembled from DNA-functionalized ultra-small iron oxide nanoparticles and further loaded with drug molecules (drug-GAN)	GSH-triggered GAN disassembly to induce efficient LPO for ferroptosis	Yue et al. (2023)
HSA@Pt(IV)	Bionic albumin shell and Pt (IV) nucleus	Affecting intracellular iron homeostasis	Tian et al. (2023)
VF/S/A@CaP	PEG2K-DSPE, 1,2 dioleoyl-sn-glycero-3 phosphate sodium salt (DOPA), and cholesterol are used to form liposome to encapsulate Vc–Fe(II) and si-OTUB2 in the core, then calcium ions and phosphate ions self-assembled on the liposome surface to form CaP shell to absorb ASO-MALAT1	Fe^2+^ released from Vc-Fe(II) generates •OH through Fenton reaction, then GPX4 is consumed to sensitize NSCLCs to ferroptosis due to the GSH depletion	Wang et al. (2023b)
MNP@BQR@ANG-EXO-siGPX4	Angiopep-2 peptide modified exosomes containing Fe_3_O_4_ nanoparticles	The synergistic triple effect of the decomposition of dihydrowhey dehydrogenase, GPX4 -ferroptosis defense axis and Fe_3_O_4_ nanoparticles mediated Fe^2+^release	Li et al. (2022d), Wang et al. (2023c)
PIOC@CM NPs	A cancer homologous targeting biomimetic nanoparticle coated with Fe_3_O_4_ and Ce6	Increasing ROS levels and consuming GSH resulting in the loss of GPX4 activity	Zhu et al. (2022)
Fe_3_O_4_-siPD-L1@M-_BV2_	SiPD-L1 is connected to Fe_3_O_4_ nanoparticles via disulfide bond to obtain Fe_3_O_4_-siPD-L1, then reacted with maleimide modified carboxyfluorescein (FAM-MALs) to prepare Fe_3_O_4_ FAM, finally M-_BV2_ is coated on the surface of Fe_3_O_4_-FAM.	Increasing the accumulation of siPD-L1 and Fe^2+^, increasing ROS, LPO, and H_2_O_2_ levels, reducing GSH levels, finally inducing Fenton reaction t	Liu et al. (2022c)
IONP@PTX	Fe (acac) 3, oleamine, and oleic acid are added to benzyl ether to synthesize oleic acid-coated iron oxide nanoparticles (IONP), then Paclitaxel (PTX) is mixed with oleic acid-coated IONP	PTX can produce ROS, IONP releases excessive Fe^2+^, ultimately producing excessive ROS and ferroptosis	Nie et al. (2023)

Nanoparticles has the ability to load chemotherapy drugs or tumor selective molecules, making them highly suitable for exploring nanoparticle-based malignant tumor treatments and combined therapies. Firstly, there is insufficient H_2_O_2_ in the acidic tumor microenvironment (TME) (Yu et al., 2023b), and a number of nanoparticles are designed to accelerate the generation of H_2_O_2_ near the tumor cell membrane (Zhang et al., 2023a), triggering Fenton/Fenton-like reaction to produce hydroxyl radicals (• OH) and ROS, amplifying the oxidative stress response (Liu et al., 2023b; Zhang et al., 2023b), and accomplishing ferroptosis within tumor cells. Secondly, overexpression of GSH in TME clears ROS and weakens the performance of ferroptosis therapy (FT) in tumors. The use of nanoparticles consumes the highly expressed GSH in tumors, which leads to the inactivation of GPX4 and accumulation of iron (Sun et al., 2023), thereby triggering ferroptosis and achieving high-performance FT.

Nowadays, extensive experiments and studies have been conducted on the combination of nanoparticles and biological compounds for treatment, but there is relatively little research on ferroptosis. DHA is a first-line antimalarial drug that acts as an inhibitor of system Xc- and GPX4. System Xc-is a heterodimeric cell surface amino acid reverse transporter protein, including transmembrane transporter SLC7A11 which is crucial in initiating ferroptosis. Based on current research, the targets of ferroptosis in lung cancer mainly focus on the regulation of antioxidant pathways, among which GPX4 and SLC7A11 are the core targets of GSH-dependent antioxidant pathways (Xing et al., 2023). On the contrary, due to the inherent drawbacks of DHA including poor stability, low water solubility, and short plasma half-life, its therapeutic efficacy is compromised. To overcome these shortcomings, nanoscale drug delivery systems (NDDSs), such as polymer nanoparticles (NPs), have been introduced to maximize the efficacy of DHA in single or multiple drug therapies (Wong et al., 2022). Fu et al. (2023) constructed an inhalable biomineralized liposome LDM loaded with DHA and pH-responsive calcium phosphate (CaP) as an iron transport nano-inducer for Fenton-like reaction to finish intracellular ROS production and ferroptosis. Zhang et al. (2023c) combined polyglutamic acid-stable Fe_3_O_4_ magnetic nanoparticles (Fe_3_O_4_-PGA-DHA) loaded with DHA with polyaspartic acid-stable Fe_3_O_4_ magnetic nanoparticles (Fe_3_O_4_-PASP-DOX) loaded with DOX to achieve significantly more intracellular accumulation of ROS and lipid peroxides for ferroptosis to enhance chemotherapy of triple negative breast cancer. Ta et al. (2023) constructed photosynthetic microcapsules (PMCs) by encapsulating cyanobacteria and up-conversing nanoparticles in alginate microcapsules which driven by external near-infrared photons. The combination of PMC and X-rays activated ferroptosis in MM cells and heterologous plants, delivering evidence for the development of lipid peroxidation, GPX4 inhibition, Fe^2+^release, and GSH reduction.

What’s more, there have been several research reports on other biological compounds associated with nanotechnology inducing ferroptosis in tumor cells. Feng et al. (2023b) reported that Fe(III)-Shikonin (FeShik) supramolecular nanomedicine could respond to the tumor microenvironment and be decomposed into Fe^2+^and shikonin, leading to immunogenic cell death through ferroptosis in tumor cells. Wang et al. (2023) constructed a BSA-FA functionalized iron-containing metal organic framework (TPL@TFBF). Through the coordination between tannic acid (TA) and Fe^3+^, a metal organic framework is formed, then loaded with Triptolide (TPL) and coated with FA modified BSA. The nanoparticles were targeted to modify tumor cells through FA, releasing TPL, Fe^3+^ and TA. After that, Fe^3+^ was reduced to Fe^2+^by TA, triggering Fenton reaction and resulting in ROS production. Additionally, TPL increased intracellular ROS production and induced ferroptosis in tumor cells by preventing the expression of nuclear factor erythroid-2(Nrf2) related factors.

## 7 Conclusion and prospects

Since ferroptosis was discovered, it has become increasingly associated with tumors and expected to deliver new avenues for anti-cancer treatments. First, some tumor suppressors, such as p53 and BRCA1-associated protein 1 (BAP1), are involved in regulating ferroptosis in cancer cells (Jiang et al., 2015; Zhang et al., 2018). Second, cancer cells require more iron to grow and have higher ROS levels compared to normal cells, which makes cancer cells more susceptible to ferroptosis (Hassannia et al., 2019; Lei et al., 2022). Third, ferroptosis has shown potential to reverse drug resistance in tumor cells (Zhang et al., 2022d). The mesenchymal cell state of cancer cells is generally thought to be related with the spread of metastasis and the acquired drug resistance (Pattabiraman and Weinberg, 2014), and this high mesenchymal cell state has been found to be dependent on the GPX4-mediated lipid peroxidation pathway, which prevents ferroptosis. Therefore, inhibition of this pathway can induce ferroptosis to reverse tumor-acquired drug resistance (Viswanathan et al., 2017). From the above, targeting ferroptosis may provide a new strategy for cancer treatment.

In this review, we summarize the current research status of some biological compounds targeting ferroptosis in the treatment of lung cancer, some of which even reduce tumor cell resistance by inducing ferroptosis. Based on the above facts, research on ferroptosis may make a significant contribution to the development of anti-cancer drugs. Meanwhile, nanomedicine-based therapies have promising results in treating tumor diseases, confirming the therapeutic potential of this cutting-edge technology. We have summarized various nanomedicine delivery strategies that have crucial therapeutic value for tumor diseases in recent years. These nanocarriers, with their unique physical and chemical properties, reach the target site and accumulate, then avoid loss and obstruction along the way, to realize the effect of treating tumors through ferroptosis.

Although ferroptosis has shown great potential in cancer treatment, there are still many unresolved issues. For example, ferroptosis has shown a strong susceptibility to various cancers since its discovery, but its dynamic regulatory mechanisms have been still lacking in research. Is there any other substance that affects the sensitivity of ferroptosis, in addition to the accumulation of iron and lipid peroxides? Besides, as the realization of ferroptosis is usually achieved through lipid peroxidation accumulation and cell membrane damage, it remains to be determined whether lipid peroxides will damage normal cells and whether there will be other potential side effects caused by ferroptosis. Next, the occurrence of ferroptosis is due to the production of ROS by excessive free Fe^2+^ under the action of Fenton reaction, which produce lipid peroxidation caused by oxidized lipids. Cuproptosis is a newly discovered programmed cell death caused by Cu^2+^ as well (Shen et al., 2023b). Finally, accumulated research has found that some biological compounds have multiple effects in controlling malignant tumors, such as inducing autophagy to prevent apoptosis of malignant tumor cells. Is this related to factors such as the dosage of traditional Chinese medicine monomers worth further investigation (Zhai et al., 2019; Wu et al., 2022). Therefore, whether other ions can also activate ferroptosis is still a direction well-worth exploring.
